# AI and climate resilience governance

**DOI:** 10.1016/j.isci.2024.109812

**Published:** 2024-04-26

**Authors:** Sara Mehryar, Vahid Yazdanpanah, Jeffrey Tong

**Affiliations:** 1 Grantham Research Institute on Climate Change and the Environment, London School of Economics and Political Science, London, UK; 2 Department of Electronics and Computer Science, University of Southampton, Southampton, UK; 3 Intensel – Climate Risk Solutions, Singapore, Singapore

**Keywords:** Natural sciences, Earth sciences, Environmental science, Environmental policy, Social sciences

## Abstract

While artificial intelligence (AI) offers promising solutions to address climate change impacts, it also raises many application limitations and challenges. A risk governance perspective is used to analyze the role of AI in supporting decision-making for climate adaptation, spanning risk assessment, policy analysis, and implementation. This comprehensive review combines expert insights and systematic literature review. The study’s findings indicate a large emphasis on applying AI to climate “risk assessments,” particularly regarding hazard and exposure assessment, but a lack of innovative approaches and tools to evaluate *resilience* and *vulnerability* as well as *prioritization* and *implementation* process, all of which involve subjective, qualitative, and context-specific elements. Additionally, the study points out challenges such as difficulty of simulating complex long-term changes, and evolving policies and human behavior, reliance on data quality and computational resources, and the need for improved interpretability of results as areas requiring further development.

## Graphical abstract

**Figure:**
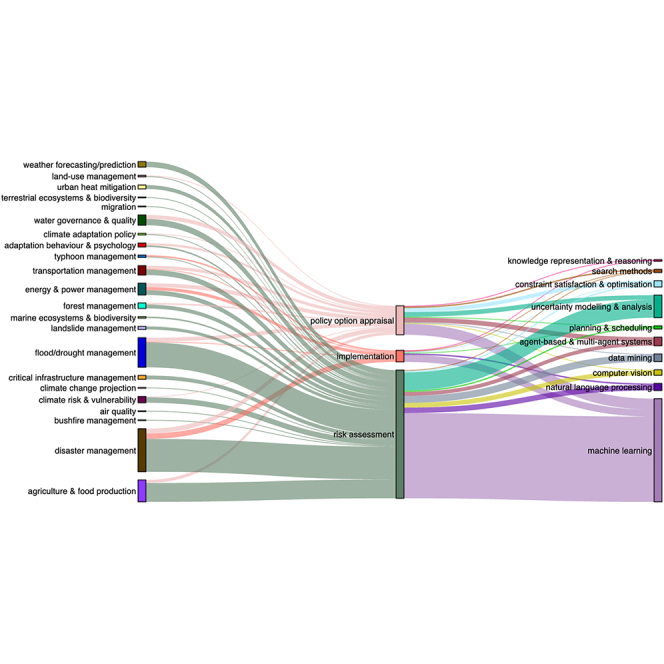

## Introduction

Climate change has emerged as one of the greatest threats facing humanity, with severe and potentially irreversible consequences for the planet and its inhabitants. In addition, climate change is rapidly increasing the frequency and intensity of natural disasters, leading to more compounding and cascading climate risks.^1^^,^^2^^,^^3^ These risks occur when multiple events, such as heatwaves, droughts, and wildfires, interact and exacerbate each other, creating a more severe and complex set of challenges. As a result, the analysis and decision-making processes for climate adaptation have become much more complicated, requiring novel methods and solutions to address these new and emerging risks.

Artificial intelligence (AI) is one such technology that can help us navigate this complex landscape by providing more advanced analytical tools. Such tools and techniques can aid in adapting to and managing climate risks by facilitating the collection and analysis of data, providing more accurate and real-time data, supporting decision-making processes, and enhancing communication between stakeholders.^4^^,^^5^^,^^6^ The utility and areas for enhancement of AI methods have been discussed in various domains related to climate change adaptation and disaster risk management. These encompass climate forecasting models,^7^^,^^8^ prediction of climate change impacts, post-disasters damage assessment, and the visualization of climate change models. Some studies have also illustrated how AI, including supervised, transferred, reinforcement, and multimodal learning methods, can leverage precise, real-time information in data-scarce settings related to climate change adaptation measures.^5^ On the other hand, several studies have recently begun to shed light on the problems and disadvantages of employing AI for climate change. Cowls et al. (2023) raise two sets of problems concerning development of AI for climate change research: the possible exacerbation of social and ethical challenges already associated with AI and the contribution to climate change through the greenhouse gases (GHGs) emitted by training data and computation-intensive AI systems. While highlighting the need for more research on the trade-offs between the GHG emissions generated by AI research and the energy and resource efficiency gains that AI can offer, they argue that leveraging the opportunities offered by AI while limiting its risks requires responsive, evidence-based, and effective governance systems. Kaack et al. (2022) introduce a systematic framework describing the positive and negative effects of AI on GHG emissions, encompassing three categories of computing-related impacts, immediate impacts of applying AI, and system-level impacts. This framework tends to support more efficient use of AI methods for climate change mitigation and adaptation. Coeckelbergh (2021) also discusses the ethical and political challenges such as violating human freedom and justice that may emerge or are exacerbated using AI for climate change.

Despite all these studies, there remains a substantial knowledge gap regarding the potential and limitations of AI in supporting governance and decision-making for climate resilience. This paper adopts a governance perspective to analyze the role of AI in climate change adaptation. It provides a comprehensive overview of how AI methods have been applied in three principal phases of risk governance including risk assessment, policy option analysis, and the implementation of adaptation policies. Additionally, we delve into the challenges and limitations, as well as the prospects and avenues for enhancing the utilization of AI methods in decision-making and governance for climate change adaptation.

Our approach, which combines a systematic literature review with an expert survey, is well suited to addressing these issues. By drawing on a diverse set of perspectives and sources, we aim to provide a comprehensive overview of the current state of AI applications in climate resilience governance and identify areas for improvement. Additionally, by supplementing our review with insights from experts in both AI and climate resilience governance, we gain a deeper understanding of the practical challenges and opportunities associated with implementing AI in this context. This will allow us to provide actionable recommendations for policymakers, practitioners, and researchers seeking to enhance climate resilience using AI.

Section 2 offers context on the climate risk governance framework and its components, as well as definitions of AI. Section 3 explains the methods employed for the analysis, i.e., systematic literature review and expert survey, detailing their implementation. Section 4 presents findings in two parts: the first part discusses the findings of the systematic literature review, elucidating how AI methods have been utilized across three stages of climate resilience governance, while the second part highlights findings from the expert survey, outlining the challenges and opportunities associated with the application of AI methods. Section 5 provides a summary of the main insights drawn from this study and explores potential pathways forward.

## Context

The term “governance” describes a model or framework for organizing and managing society.^9^ In a general term, it compromises the mechanisms, processes, and institutions, through which citizens and groups choose and implement the right set of actions to meet the needs of society.^10^^,^^11^ Climate resilience governance involves the translation of the core principles of governance to the context of resilience and adaptation to climate change. It refers to the processes and procedures through which governments, organizations, and communities plan, implement, and coordinate actions to build resilience and adapt to the impacts of climate change.^12^ It involves the development and implementation of policies, strategies, and institutional mechanisms that aim to enhance the ability of societies and ecosystems to withstand, recover from, and adapt to climate-related shocks and stresses.^13^

### Climate resilience governance framework

**Figure 1:**
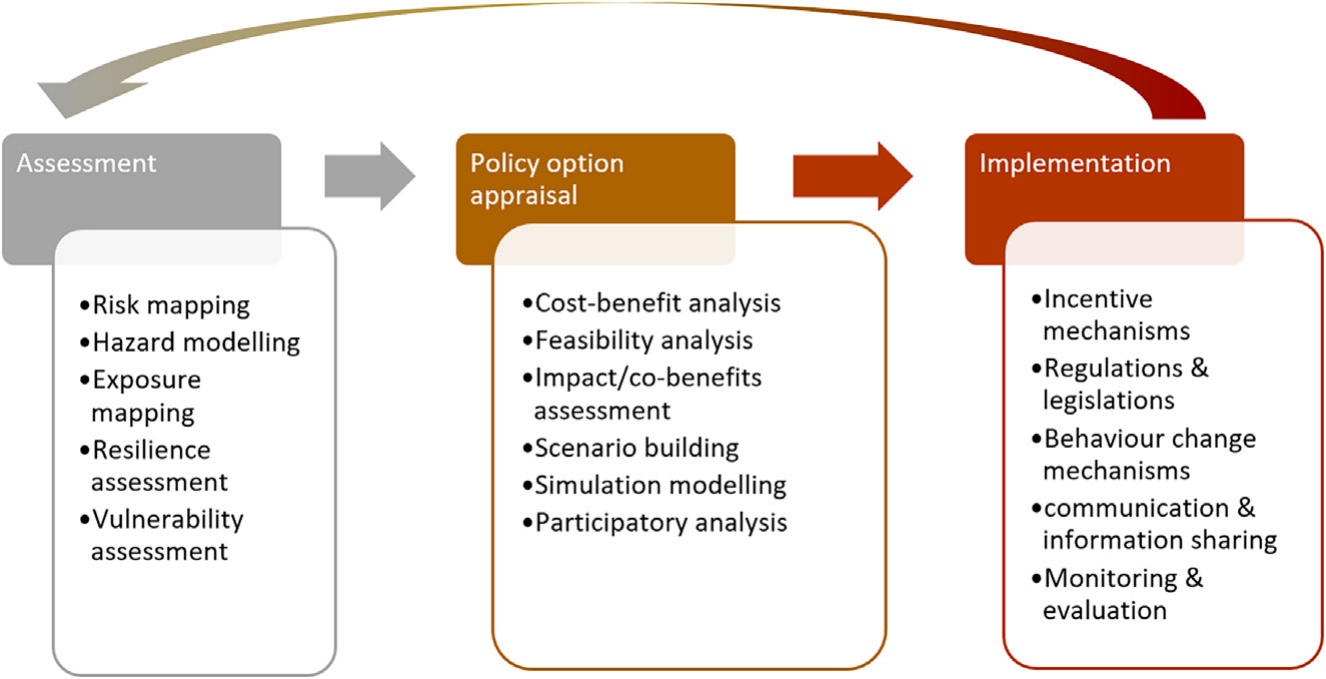
Three stages of climate resilience governance and their components The three stages are a simplified version of the UKCIP decision-making framework for climate change risk (Willows et al., 2003).

Climate resilience governance typically involves several stages or phases that are essential in decision-making. Figure 1 presents the three main stages commonly associated with climate resilience governance.

#### Risk and resilience assessment

This stage involves conducting comprehensive assessments to identify the risks and resilience associated with climate change. It includes modeling probability and severity of hazards caused by climate change (e.g., flooding, heatwaves, drought, etc.), mapping exposure to hazards, and assessing the vulnerability and resilience of populations, infrastructure, ecosystems, etc. This analysis supports estimating the potential climate change impacts and risks across sectors and locations.

#### Policy option appraisal

This stage involves evaluating different policies, strategies, measures, interventions, and approaches to enhance climate resilience. It includes identifying a range of potential actions to address climate risks; assessing the feasibility, effectiveness, costs, and benefits of each option; and finally prioritizing and selecting the preferred strategy. The decision support tools and methods used to support option appraisal include scenario building, simulation modeling (including human behaviors simulations), and participatory analysis methods.

#### Implementation

The implementation stage focuses on translating the chosen strategies into concrete actions and results. It involves planning, executing, and monitoring the implementation of resilience measures. Incentive mechanisms (such as taxes and subsidies), regulations and legislations, behavior change mechanisms, and monitoring and evaluation are among the key implementation mechanisms/tools used for climate resilience governance.

This is an iterative process as learnings from the implementation should feed into the assessment and option appraisal process.

### Artificial intelligence

In this work, we refer to AI in a broad sense as computational technologies and tools with some level of autonomy and adaptability able to reproduce intelligent abilities to undertake complex tasks like “learning” and “problem solving,” with minimal to no human intervention.^14^^,^^15^ While AI had previously been used to describe “machines that mimic human mind, and therefore, think and act like humans,” this definition has since been modified to “machines that think and act rationally, and therefore, take actions to maximize the chance of achieving pre-defined goals.”^16^ Using this approach, AI aims to develop tools and methods to perform tasks that typically require human intelligence. Since the 1940s, when scientists started discussing the possibility of creating a thinking machine, AI research has influenced human life in many ways. It has particularly revolutionized numerous sectors and research areas in recent years with its ability to process vast amounts of data, learn from patterns, and make autonomous decisions while it still has the potential to drive unprecedented progress and innovation.

More recently, AI research became interested in, and realized the necessity for, keeping humans in the loop,^17^ to capture preferences of citizens and end-users,^18^ and the fine-tuning of AI tools based on human feedback. This is to realize that advanced AI technologies are human-beneficial if they solve problems human communities are facing and in consideration of their needs and contextual values. In this regard, adapting to climate change and governing resilience in communities are crucial problems in need of effective decision support tools. This study surveyed the body of research on this topic and interviewed stakeholders, aiming to elaborate on where AI research and developed tools are actively supporting resilience governance and where there are gaps and the need for design and development of novel AI-based techniques for supporting resilience in communities.

## Methods and data

We conducted a systematic literature review (Lacey et al., 2011) of studies that applied AI to support climate resilience governance as per the three stages of governance, i.e., assessment, option appraisal, and implementation. To ensure inclusion of all relevant studies, we used a combination of generic keywords, particularly for the resilience governance part, and excluded those that were not relevant after reviewing the focus and objective of studies (e.g., application area of AI methods) mentioned in the abstracts.

**Figure 2:**
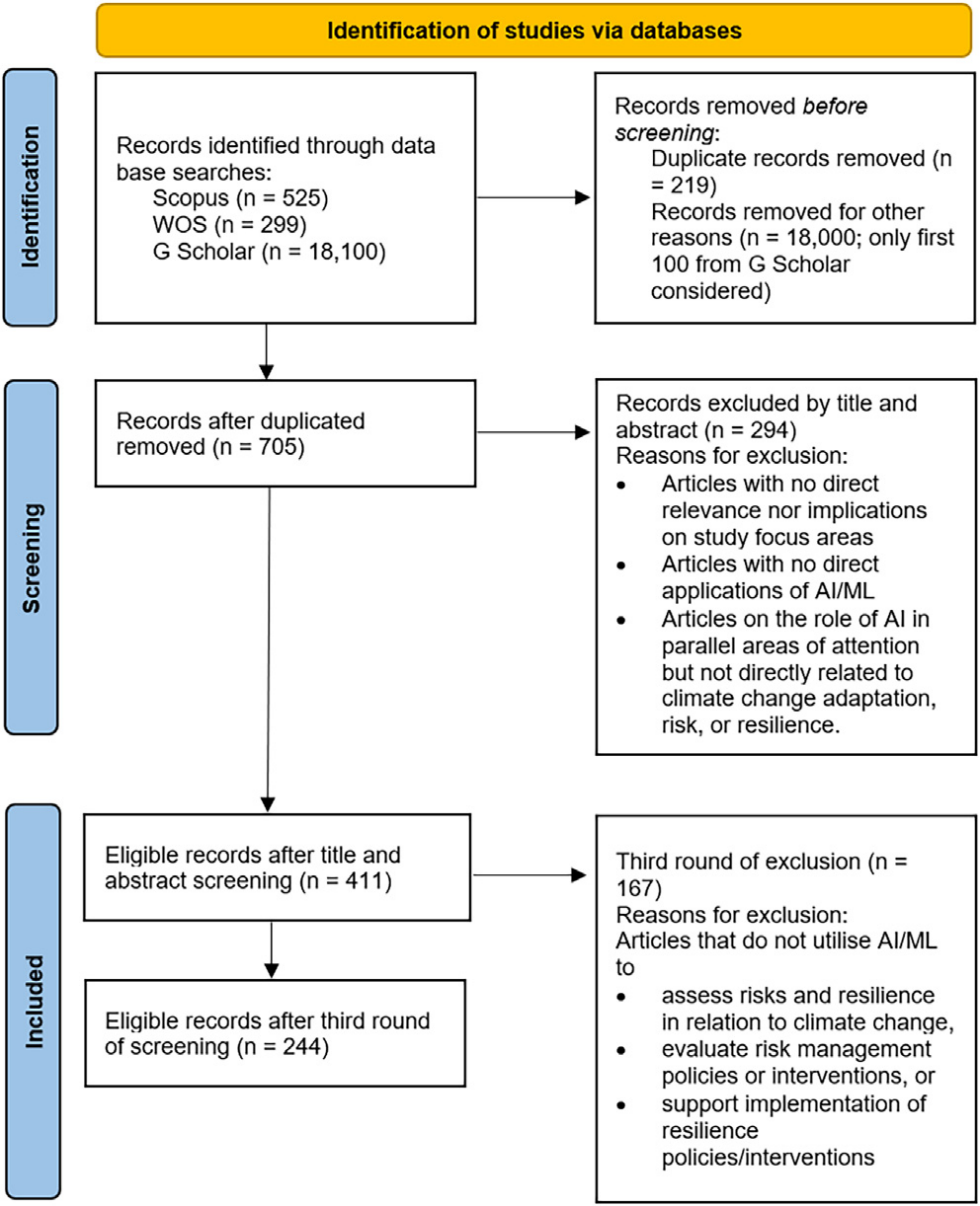
Identification, screening, and inclusion criteria for studies according to PRISMA framework

To obtain all relevant studies prior to the identification stage, we utilized a common search string within each of the three databases Scopus, World of Science, and Google Scholar (“artificial intelligence” OR “machine learning” AND “climate” OR “disaster” AND “resilience” OR “adaptation”). For each article we obtained their title, abstract, keywords, year of publication, and journal source. We then screened the articles for duplicates and excluded articles based on criteria such as relevance across three rounds as elucidated in Figure 2.

In addition, we conducted an online survey (Presser et al., 2004) utilizing purposive sampling technique (Tongco, 2007), involving experts from academia and professionals in the field of AI and/or climate change/disaster risk management. The aim was to gather experiential data regarding the utilization of AI methods by practitioners, policy makers, and researchers. The survey questioner, developed in Qualtrics, was distributed through email lists associated with AI, computational methods, climate change, or disaster risk management (see the supplementary document for the mailing lists used), as well as the network of practitioners engaged with this project, i.e., the Zurich Flood Resilience Alliance and UTU.

The survey results were instrumental in complementing the findings of our systematic review. While the literature review provided insights into taxonomies of AI applications in climate risk governance, the expert survey served to enhance our understanding on the challenges, limitations, and opportunities associated with the application of AI in risk governance, which are less elaborated in academic papers.

The overarching survey questions were as follows:

(1) What were the advantages of applying AI/ML methods in your work/research that could not be achieved otherwise?
(2) What are the challenges or limitations that hamper the use of AI/ML methods for climate change adaptation and disaster risk management?
(3) What are the opportunities or areas for improvement in applying AI methods to support climate change adaptation and disaster risk management?

The survey data were collected in May and June 2022, and the survey questions were approved by the research ethics committee of the London School of Economics and Political Science (ref. 85277). The survey questions, demographic details of the survey respondents, and data collection sources can be found in supplementary document.

## Synthesis

Two hundred forty-four articles were identified as relevant to application of AI for climate resilience governance, according to the guidelines of our research. We further categorized the 244 articles by domain, AI technique utilized, publication year, and journal source.

The number of publications addressing climate risk and resilience with AI techniques has increased in the past five years, with a high of 57 publications in 2020. These publications include journal articles, book chapters, conference papers, and report. As one of the inclusion criteria for the review was “application of an AI method,” the extracted dataset does not include any literature review papers or theoretical and conceptual papers.

The top five domains that utilized AI methods the most are disaster management (28%), flood or drought management (16.0%), agriculture and food production (15%), transportation and critical infrastructure (12%), and water governance and quality (7%), while 16 total domains were identified. In addition, the studies were categorized based on the objectives or type of analysis for which the AI methods are utilized. For example, the top research objectives/analysis are (1) climate or disaster risk modeling, (2) assessing impact of climate change on crops, (3) climate change adaptation planning, (4) response and recovery planning, and (5) social media analysis.

**Figure 3:**
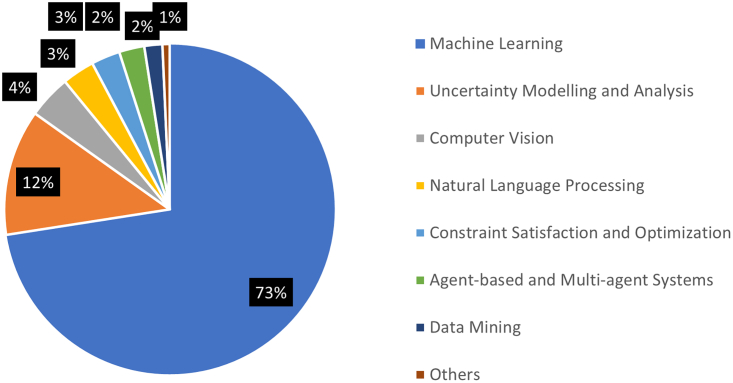
Percentage of publications by AI techniques employed

The articles were also coded based on the specific AI methods used and then were categorized into 15 broad categories of AI methods. As shown in Figure 3, a significantly large number of studies employed machine learning as the main AI method (53% of studies), followed by uncertainty modeling and analysis (11%), data mining (4%), natural language processing (4%), and agent-based and multi-agent systems (4%).

### Application of AI in climate change studies based on the three stages of climate resilience governance

**Figure 4:**
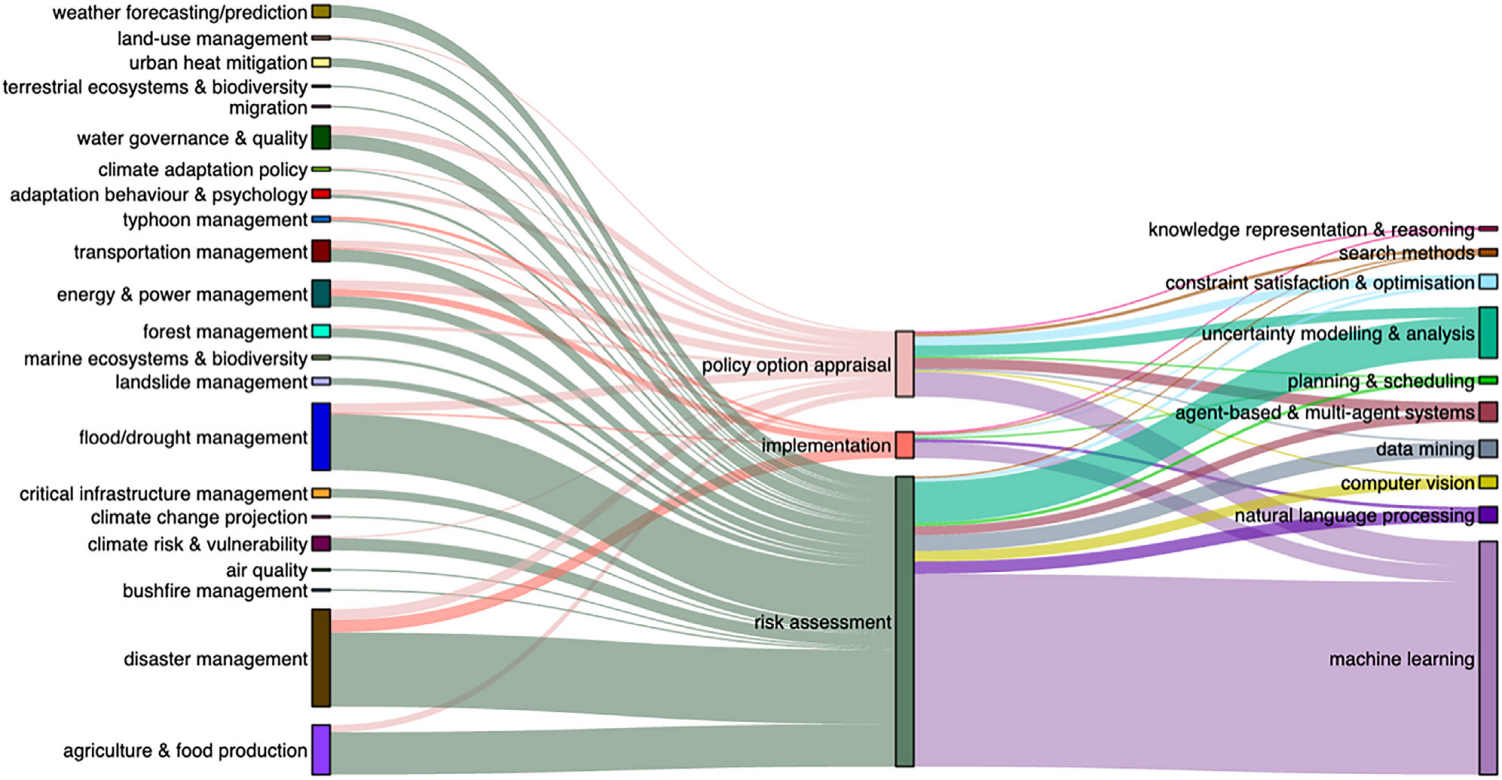
Visual representation of studies categorized by application domains, resilience governance categories, and AI techniques employed

Based on the framework introduced in sections 2, each study was classified according to its main objectives and research questions among three categories: assessment, policy option appraisal, and implementation. Where a study has been identified as addressing two or more goals (e.g., covering both assessment and policy analysis), their most dominant focus area is taken. Figure 4 illustrates the distribution of studies across different categories of governance, AI techniques, and application domains. A significant portion of the studies is dedicated to risk assessment, employing machine learning tools to enhance support for disaster management.

#### Risk and resilience assessment

**Figure 5:**
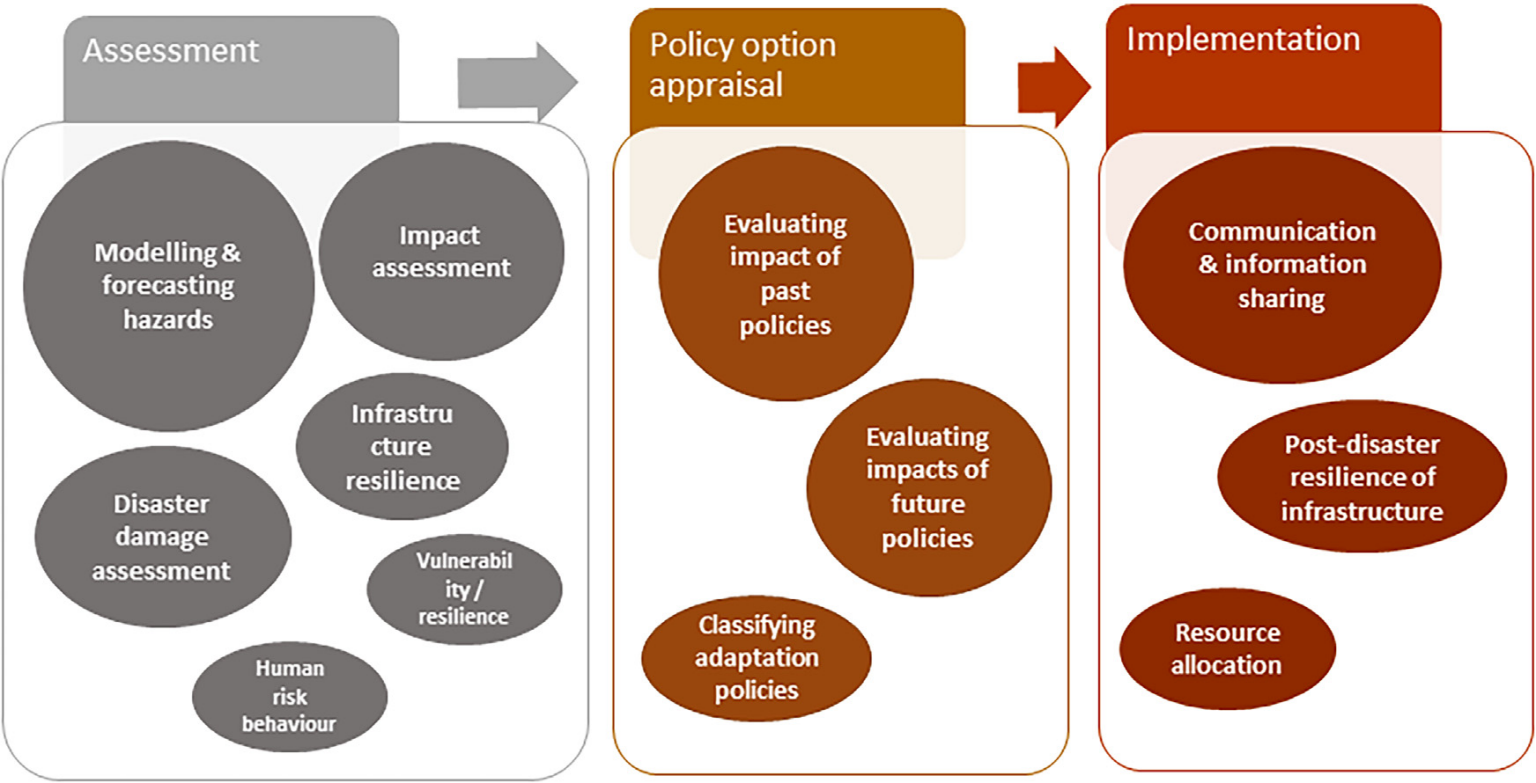
How AI is applied in climate change adaptation studies, categorized in three stages of climate risk governance.

A large proportion (64%) of studies analyzed focus only on risk and resilience assessment. Most common application domains associated with “assessment” category are (1) disaster management, (2) flood/drought management, and (3) agriculture and food production. These include studies on assessing the following (Figure 5):

(1) *Climate/disaster risks modeling* (24%): this involves modeling and forecasting climate-related hazards as well as modeling future vulnerability and exposure of infrastructures, buildings, populations, and ecosystems. Typical risk modeling studies observed includes applying machine learning techniques to improve accuracy of long-term weather forecasting and hazard mapping for drought,^19^ rainfall/flooding,^20^^,^^21^^,^^22^^,^^23^^,^^24^ heatwave, and water scarcity.^25^ These studies also incorporate to an extent uncertainty modeling and analysis techniques.
(2) *Impact of climate change and extreme events*: this is assessing long-term impact of climate change scenarios on crop production, water resources, infrastructures, and communities’ vulnerability.
(3) *Disaster damages*: this includes post-disaster assessment of damages on buildings and infrastructures mainly to inform emergency response and relief sectors.
(4) *Infrastructure resilience*: this includes evaluating and predicting resilience of infrastructures facing disasters and identifying the vulnerable infrastructures.
(5) *Community vulnerability/resilience*: this category of studies evaluates the vulnerability or resilience of communities mainly based on the information from the past events.
(6) *Human risk behavior*: a few studies have also been found that investigate human risk-related behavior (decisions and actions) in the past events, such as mobility pattern and households’ recovery patterns.

*AI methods used:* a large proportion (>50%) of papers categorized as “assessment” studies are associated with the application of machine learning as the main AI technique. Specific machine learning techniques applied the most include random forests, artificial neural network, deep/reinforcement learning, and support vector machine. In climate risk modeling, such methods are often used to analyze and evaluate probabilities of scenarios or relationships between interrelated variables to classify areas based on the expected likelihood of occurrence of hazards. In addition, AI techniques are often used to analyze multiple forms and types of data derived from computational approaches such as remote sensing, social sensing, or crowd sourcing technologies. The most used computational approaches in risk and resilience assessment are described in Table 1.

**Table 1:** Common computational techniques for risk/resilience assessment

Computational technique	Example of application
Remote Sensing	ML is often used to extract data from remote sensing data (e.g., via image segmentation, object detection, and tracking) to identify differences in environmental infrastructure or land use/land cover.
Social Sensing	Use of natural language processing to analyze social media information, disaster phenomena, or spatiotemporal correlations between infrastructure and societal impacts. Such techniques collate and classify information for more efficient interpretation by practitioners or decision-makers (e.g., classifying post-disaster twitter data and images for damage assessment)
Crowdsourcing	Combination of human and machine intelligence, e.g., human-in-the-loop to improve algorithms.
Dynamic Network Analysis	Increase visibility of relationships among entities or heterogeneous actors, e.g., via meta-network analysis, knowledge graphs
Probabilistic Forecasting	Elicitation of magnitude and probabilities of hazard (e.g., flooding and inundation) allowing for quick discrimination of safe and exposed areas
Simulation	Use of agent-based, statistical models and/or cellular automata to analyze individual behaviors and interactions in relation to the evolution of land-use change and their impacts
Ensemble Models	Ensemble models analyze relationships between interrelated variables and classification of areas based on expected likelihood of occurrence of hazard. Increasingly ensemble or hybrid machine learning employed (e.g., REPTree, Naive Bayes, bagging, random subspace) for high-precision flood susceptibility models.

#### Policy appraisal

Twenty-nine percent of studies analyzed apply a kind of AI method to analyze various policies, measures, and interventions, among which 5% include both risk assessment and policy option analysis in one study. Most common application domains associated with “policy appraisal” category are water governance and quality followed by disaster management. These studies conduct policy option analysis by (Figure 5):

(1) *Evaluating impact of policies in the past*: these studies take a backward-looking analysis approach and mainly use machine learning techniques in image processing^26^^,^^27^^,^^28^ or analyzing socio-economic survey data^29^ to evaluate impact of adaptation and risk reduction policies on the physical, social, and economic recovery of communities following a disaster.
(2) *Evaluating potential impact of policies before their implementation*: these studies use either modeling or participatory approach to evaluate and compare potential impacts of different policies. Modeling and simulation approaches in these studies often consider cost-benefit analysis of actions^30^^,^^31^^,^^32^^,^^33^^,^^34^^,^^35^ or optimization of the speed of recovery^36^^,^^37^^,^^38^^,^^39^ in analyzing impact of policies, while participatory approaches often take account of a diverse range of criteria including interests, values, and preferences of stakeholders in analyzing impact of policies.^40^^,^^41^ Agent-based models have also been used to simulate impact of policies and strategies considering human behavior including human beliefs, expectations, and values in decision-making.^42^^,^^43^^,^^44^.
(3) *Identifying and classifying adaptation strategies*: there are a few numbers of studies (i.e., 2%) that focus on collecting and showcasing adaptation strategies and measures from various case studies.

*AI methods used:* in many studies, the same methods and tools utilized in earlier risk assessments form the basis for subsequent policy options and scenarios. However, in moving from risk and resilience assessment to policy goals, studies may also integrate analyses of prevailing socio-economic conditions, thus recognizing the multivariate and multidimensional nature of vulnerability and resilience.^45^ Like the risk and resilience assessment, machine learning has been used in majority of the studies related to policy analysis. ML methods are often being used to identify and evaluate alternative policies and actions as part of a broader decision-support system (DSS). Florez et al. (2015), for example, utilize AI approaches (multi-stages stochastic program) to identify optimal locations for the placement of a humanitarian facility/warehouse within a decision-making framework, taking into consideration multiple factors including road networks, occurrence of disaster, warehouse capacities, and transportation costs. In a case study of optimizing agricultural land use in Victoria, Australia, Sposito et al. (2010) develop a decision-making framework integrating land suitability analysis with uncertainty analysis and spatial optimization to enable practitioners to identify resilient alternatives to cropping systems and to investigate policy actions to adapt to the impacts of climate change. Agent-based modeling (ABM) has also been employed in simulating possible impacts of policies. Ghaffarian et al. (2021), for example, conducted behavior modeling of different population groups to analyze the impact of site relocations on the employment rates among formal and informal settlement households in post-disaster recovery.

#### Implementation

A small proportion of studies analyzed (i.e., 6%) apply an AI method to support implementation of adaptation and resilience actions. Most common application domains associated with “policy appraisal” category is “energy and power management.” Such studies mainly focus on the following (Figure 5):

(1) *Improving communication and information sharing*: among these are studies that use natural language processing to either connect user input to relevant knowledge discovery channels in the post-disaster communications—via smartphone application, web-based systems, and smart home devices^46^—or automate planning models for disaster response and recovery activities.^47^ Some studies also use machine learning approaches to collect, integrate, and analyze data in order to improve communication and decision-making among humanitarian relief actors^48^^,^^49^ and timely access to critical information of resources and services.^50^
(2) *Improving resource allocation models*: this includes studies that develop a type of decision support system for emergency operations to support efficient and fair allocation of limited resources following a disaster, e.g., automation of planning models.^51^^,^^52^
(3) *Improving post-disaster resilience of infrastructure*: these studies often focus on using machine learning techniques to improve resilience and resistance of infrastructures such as power supply, sensor web systems, and transportation networks against future disasters.^53^^,^^54^^,^^55^^,^^56^

### Application of AI by users; opportunities and challenges

The expert survey on the application of AI in climate change adaptation and disaster risk management received 40 valid responses. Most respondents (70%) are from the academia/research sector, followed by non-profit/NGOs (12.5%), the private sector (12.5%), and the government sector (5%). Their areas of expertise and research focus encompassed a wide range of fields, with climate change adaptation/resilience and disaster risk management being the most indicated areas of expertise. While 37.5% of responders indicated they have moderate familiarity (rating 2 on a scale of 1–5) with AI/ML, 17.5% considered themselves very familiar (rating 5). In addition, 60% of respondents reported having employed AI/ML methods in climate change adaptation and disaster risk management, with agent-based models, neural networks, and unsupervised learning being the most used methods, and 40% of the respondents stated that they had not directly used such methods—demographic information of the respondents can be found in supplementary 1.

We supplemented the results of the survey with opportunities and challenges found in the systematic literature review to elaborate on and provide a more comprehensive understanding of the subjects.

#### Advantages and opportunities

Respondents highlighted several advantages of applying AI methods in their work. The most important advantages mentioned by many respondents is the ability to handle *complex and multi-variable problems* as well as *non-linear relationships* that are not amenable to statistical or classical approaches. The second most important advantage (which is also related to the first one) is *processing large quantities of data* (particularly multidimensional datasets) efficiently. Other benefits, mentioned by fewer number of respondents but still quite important, were pattern recognition, the ease of comparing different approaches, and predicting trends and future scenarios to support decision-making.

The latter has also been highlighted in the literature as advantages of AI that can be further explored and utilized. It has been discussed that computational approaches have the ability to model potential effects of policy scenarios and trajectories^57^^,^^58^ and account for their uncertainties. They may (1) account for potential new information learned in the future (non-stationary, stochastic, dynamic) to enable the further evaluation of options (e.g., cost-benefit analysis, weigh flexibility/redundancy vs. efficiency, etc.), (2) model future behavior (e.g., simulation of post-disaster recovery processes), and (3) quantify future learning in adaptive policies and the effectiveness of flexible planning.^58^ In addition, AI and machine learning techniques may enable optimal policy formulation independent of the past. Nozhati et al. (2020) describe how the use of Dynamic Programming (DP) may capture trade-offs between the present and the future, ordering decisions based on their “sum of the present cost (or reward) and expected discounted future costs.” Similarly, reinforcement learning techniques do not require prior knowledge of communities and may be suitable in the absence of a model.^38^

#### Challenges and limitations

Despite the potential benefits, several challenges and limitations were identified in the use of AI methods for climate change adaptation and disaster risk management. The predominant challenges identified by numerous participants revolved around lack of expertise, capacity, and knowledge in both AI and climate change sciences within organizations. Therefore, the lack of organizational capacity in integrating these two fields seems to be a persistent gap in applying AI for climate change projects. However, a smaller subset of participants outlined challenges and limitations with relatively high significance in terms of areas for improvement. While these may be seen as outlier opinion among the participants of this study, they were acknowledged in the literature and worth further consideration. We categorized these into five overarching themes of challenges and limitations.

(1) *Failing to simulate long-term complex changes over time*: climate change models face challenges in simulating the complex and dynamic evolution of real-world systems over time. Ecosystem and land-use changes often unfold over decades, while models struggle to accurately reflect shifting biophysical dynamics, demographics, and land-covers.^21^^,^^59^ Furthermore, both natural and human systems exhibit inherent randomness, stochastic dynamics, emergent behaviors, and nonlinear feedback that are challenging to fully represent in models. For example, the exact timing and location of severe storms cannot be predicted deterministically as simulation models do not often encompass all uncertainty sources.^60^ Therefore, models struggle to accurately simulate how shifting climate may impact complex ecological connections or cascade through social systems in unexpected ways. Advanced techniques help but uncertainties remain.
(2) *Failing to account for changes in policies, decisions, and human behavior*: climate models often optimize decisions based on isolated points in time, disregarding the potential for technological advancements, shifts in societal preferences, and increasing resilience over time^33^^,^^59^. Similarly, decision support tools and models tend to optimize adaptive measures solely on present climate exposure and vulnerability, neglecting the fact that both exposure and vulnerability can evolve over time due to external events or the impacts of new policies and behavioral changes. Although some newer multi-stage models attempt to address some of these dynamics by optimizing over multiple time periods, most models still fall short in fully accommodating the progressive growth in ambition, policy impacts, and decision-making that is likely to unfold in the future. Consequently, these challenges can render the models as static snapshots rather than adaptive simulations, possibly leading to a conservative estimate of the pace of future climate progress. Effectively capturing the potential for future improvements in climate action and resilience remains a significant computational modeling challenge.
(3) *Climate assessment modeling and simulation is constrained by the quality of input data and high computational demands*: model accuracy is heavily dependent on the coverage, granularity, and quality of input data. For example, climate models rely on weather station data to calibrate and validate simulations. Sparsely distributed or short historical records limit model performance.^61^ Scarcity of training data is more problematic for extreme events with low probability. Additionally, incorporating higher resolution datasets requires greater computational power and optimization methods. Advancing climate insights involves an ongoing need to improve monitoring and data integration while scaling computational capabilities and efficiency.
(4) *Insufficient data on socio-economic indicators that shape climate vulnerabilities and resilience*: climate risk assessments using AI often focus narrowly on analyzing “hazards,” like probability and severity of extreme weather events, and to a lesser degree on “exposure” while assessing vulnerability as the third component of creating risk (as risk = hazard∗exposure∗vulnerability) is often overlooked in such assessments. This is largely because assessing vulnerability and resilience concerns subjective, qualitative, and context specific factors such as inequality, risk awareness, social network, and risk perceptions, which presents challenges for AI. AI algorithms, which reply heavily on quantitative data and patterns, struggle to capture nuanced and subjective aspects inherent in vulnerability and resilience assessment. Even when proxies like income are used, inherent uncertainties exist in relating proxies to the underlying multidimensional variables of vulnerability or resilience. Moreover, the interrelatedness of these factors further complicates AI analysis, potentially leading to oversimplification or misinterpretation of complex situations. Inequality, a crucial dimension of vulnerability, can be perpetuated or exacerbated by AI algorithms due to biases in training data. Adequate representation of the socioeconomic contexts conditioning climate risk models may misestimate or mis-optimize decisions. Advancing techniques to integrate disparate or qualitative socioeconomic data streams and better encapsulate their relationships with physical climate projections remains an ongoing modeling challenge.
(5) *Interpretability of results and uncertainties in translating outcomes of analysis to implementation:* the complex, multidimensional nature of climate models makes their outputs inherently challenging for even experts to fully comprehend and clearly interpret. These interpretability constraints propagate uncertainties when translating model outputs into climate actions and policies.^60^ Findings require expert judgment and on-the-ground investigation to determine appropriate interventions based on specific regional factors and community vulnerabilities. Complementary processes are essential to bridge broad model insights with situated decision-making tailored to local needs. Advancing AI models’ explainability and expert-guided contextualization remains critical to enable actionable insights.

In addition, the moral and ethical implications of AI applications were also raised as the practical challenges of applying AI methods. Participants notably highlighted the indispensable role of human judgment and knowledge, emphasizing that certain aspects cannot be replaced by AI/ML algorithms.

The respondents also identified application areas in which AI methods and techniques can be used at a greater scale or frequency. These application areas are operational disaster risk management (60% of total responses), climate vulnerability and resilience (50%), transportation and critical infrastructure (45%), risk finance (45%), and agriculture/food production (32%). The participants elaborated on the need for new technologies like AI to help understand emerging phenomena in these fields. Preparation for uncertain future events requires scenario building, for which ML could be useful. In addition, as problems in these fields become more complex, scientific knowledge falls short in addressing them fully. AI techniques can analyze interdependencies between components and systemic issues that result from such interdependencies.

## Conclusion and discussion

This study unveils a multifaceted landscape of AI applications in climate risk governance, encompassing risk assessment, policy analysis, and implementation. It highlights that a substantial portion of research predominantly focuses on enhancing methodologies for “risk assessment” while there has been much less effort in supporting “policy evaluation” and “implementation.” Machine learning techniques like random forests, artificial neural networks, and deep reinforcement learning find widespread use across various domains, including probabilistic forecasting simulation, remote sensing, image processing, social sensing, dynamic network analysis, and ensemble modeling. These techniques aid in modeling climate-related hazards, forecasting extreme weather events, assessing climate change impacts, evaluating infrastructure resilience, and analyzing human risk behavior. However, AI has predominantly been applied to *hazards* and *exposure* analysis in support of risk assessments, with less emphasis on “*resilience* and *vulnerability* assessment,” primarily due to the scarcity of reliable socioeconomic data for measuring community resilience and vulnerability to climate hazards. Furthermore, while some studies employ AI for decision support and policy analysis, they often focus on evaluating the retrospective impacts of implemented policies. There is a need for more research to simulate and measure potential policy impacts, including co-benefits and maladaptation consequences, as well as system responses before policy implementation, facilitating comparisons and prioritization. Human behavior, including perceptions, values, priorities, decisions, and actions, is also an important element of risk assessment, policy analysis, and adaptation implementation. Despite the recent advancements, AI tools still face challenges in modeling and predicting human behavior due to its high subjective, complex, and dynamic nature. However, AI techniques like machine learning can uncover complex patterns and relationships within large datasets, helping researchers in understanding the drivers of human behavior related to climate change adaptation. Furthermore, AI enables real-time feedback to individuals and communities regarding their behavior’s impact on adaptation, and personalized interventions based on individual preferences, attitudes, and behavior patterns, thereby enhancing evaluation and prediction of adaptation strategies. Integrating these AI techniques with approaches formalizing human behavior or translating qualitative data into quantitative data could advance AI applications for human adaptation behavior.

The study also draws insights from an expert survey, emphasizing the promising advantages of AI in practical terms, such as tackling complex, multi-variable problems and efficiently processing large datasets, thereby enabling trend prediction and informed decision-making. However, persistent challenges in applying AI to climate change adaptation and disaster risk management exist. Firstly, simulating long-term climate changes and their impacts on human behavior and infrastructure is complex, requiring sophisticated modeling techniques to capture and predict their evolution accurately. Moreover, AI models must adapt to the evolving landscape of policies and societal attitudes, necessitating continuous refinement to provide relevant and actionable insights over time. Ensuring data quality is another critical challenge in leveraging AI for these purposes, given the uncertainties and biases inherent in environmental and social datasets. Careful attention must be paid to data collection, validation, and preprocessing to mitigate potential inaccuracies and biases that could undermine the reliability of AI-driven analyses and predictions. Additionally, the interpretability of complex AI models emerges as a key concern. Understanding how these models arrive at their decisions is crucial for building trust and facilitating meaningful engagement with stakeholders. Enhancing the transparency and interpretability of AI models is essential to foster confidence in their outputs and ensure their responsible use in decision-making processes.

Furthermore, while AI offers unprecedented computational power and analytical capabilities, the continued importance of human expertise cannot be overstated. Expert judgment and domain knowledge remain indispensable for contextualizing AI-driven insights and translating them into actionable strategies and policies. Finally, ethical considerations also loom large in the application of AI to climate change adaptation and disaster risk management. Addressing concerns such as algorithmic bias and privacy issues is crucial to safeguard against unintended consequences and ensure equitable outcomes.

As a direction to extend this work, there is a need for studies on the application of AI in specific pressing domains such as heatwave resilience and health as well as emerging compounding and cascading climate risks. We believe advancements in AI can support these time-demanding concerns and help communities’ resilience toward potential epidemics. To that end, AI tools need to intergrade lessons learned from the COVID pandemic and investigate more effective resource allocation techniques (for which there exists a large body of theoretical work rooted in computational game theory). The other direction that could be further explored is generative AI models and foundational models for understanding human-level communications (e.g., ChatGPT and similar tools). Such tools have the potential to operate in multimodal domains, e.g., by taking geographical images and videos as input, and provide guidelines, early signals, and community alerts using a variety of sources. Indeed, how to monitor such tools and keep them under human oversight is an open problem. In general, we see high potential for interdisciplinary research on AI for resilience governance and invite these research communities to work on developing tools for building a more sustainable society.

### Limitations of study

As a caveat, it should be noted that in this study we opted to focus solely on articles explicitly mentioning the term “artificial intelligence” at least once to maintain consistency and clarity in analysis. We acknowledge that there may exist articles that employ various AI methods without explicitly using the term “artificial intelligence.” Nonetheless, we believe that the results and findings presented in this study are representative of the broader landscape of AI-related studies in the context of climate change and disaster risk management.

## Methods

### Key resources table

**Table:** 

REAGENT or RESOURCE	SOURCE	IDENTIFIER
**Software and algorithms**		

Microsoft Excel	Microsoft	
SankeyMATIC	sankeymatic.com	

### Resources availability

#### Lead contact

Further information and requests for resources should be directed to and will be fulfilled by the lead contact, Dr. Sara Mehryar (s.mehryar@lse.ac.uk).

#### Material availability

This study did not generate new unique reagents.

#### Data and code availability

All data used to perform analyses have been included as publicly available supplemental information. Any additional information required to reanalyse the data reported in this paper is available from the lead contact upon request. This paper does not report original code.

### Method details

A combination of systematic review and expert survey has been applied in this study. The database collected for the systematic literature review together with the data analysis were documented on Open Science Framework (https://osf.io/6rjk2/) on April 21, 2024. The NIH and ARRIVE reporting guidelines for preclinical work do not apply. All methods are detailed in our manuscript and supplemental files.

## Supplemental information

Document S1. Summary of survey results
