# Supplementary material for: AI and climate resilience governance
Source: iScience. 2024 Apr 26;27(6):109812. doi: 10.1016/j.isci.2024.109812 (PMC11112607; doi:10.1016/j.isci.2024.109812)
Supplement: Document S1. Summary of survey results [file mmc1.pdf]

iScience, Volume *27*

Supplemental information

AI and climate resilience governance

Sara Mehryar, Vahid Yazdanpanah, and Jeffrey Tong

Summary of Survey Results

- *40 valid responses*
- *The majority group highlighted in yellow.*

Demographic questions

1. In which sector do you currently work?
    a. Academia/Research: 28 (70%)
    b. Government: 2 (5%)
    c. Non-profit/NGO: 5 (12.5%)
    d. Private Sector: 5 (12.5%)
2. Please indicate your areas of expertise, responsibility, or research focus (e.g., operational disaster risk management, risk finance, transportation, climate adaptation, AI & Machine learning, supply chain management, etc.).

**Area Indicated**	**No. of respondents**	**% respondents**
AI and Machine Learning Research	2	5
Business continuity, Safety management, Emergency response	1	2.5
Climate Change Adaptation/Resilience	6	15
Climate Policy	2	5
Computational Geography	1	2.5
Cybersecurity	1	2.5
Disaster Risk Management/CCA	10	25
Extreme Events/Hazard Modelling	3	7.5
Hydro(geo)logical Modelling	1	2.5
Operational Meteorology	1	2.5
Malaria and public health	1	2.5
Supply chain management	1	2.5
Risk analysis and finance	6	15
Seismic and multi-hazard	1	2.5
speech signal processing, machine learning, NLP	1	2.5
STS	1	2.5
Urban adaptation planning	1	2.5
Total	**40**	**100**

3. For how long have you been working in this/these fields?
    a. Less than 3 years: 4 (10%)
    b. 3-10 years: 16 (40%)
    c. 10-20 years: 12 (30%)
    d. More than 20 years: 8 (20%)
4. Gender
    a. Female: 12 (30%)
    b. Male: 26 (65%)

c. Prefer not to say: 2 (5%)

Research specific questions

5. On a scale of 1 to 5, with 1 being ‘not familiar at all' and 5 being ‘very familiar’, how would you describe your familiarity with the topic of AI/ML?

Option	No. of respondents	% respondents
1 - Not familiar at all	3	7.5%
2	15	37.5%
3	5	12.5%
4	10	25%
5 - Very familiar	7	17.5%

6. Have you ever used an AI/ML method in your work/study related to climate change adaptation and disaster risk management? If yes, what was that? (please select all that apply)

	Number of respondents who selected option	% respondents
1. I have never used an AI/ML method in my work/study	16	40
2. Agent-based models	11	27.5
3. Random decision forests	8	20
4. Support vector machines	6	15
5. Neural networks	7	17.5
6. Boosted regression trees	7	17.5
7. Generalised linear models	6	15
8. Bayesian methods	10	25
9. Natural language processing	7	17.5
10. Maximum entropy	3	7.5
11. Reinforcement learning	2	5
12. Supervised learning	9	22.5
13. Unsupervised learning	10	25
14. Fuzzy methods in decision-making	5	12.5
15. Others (response: “not sure what is used”)	1	2.5

7. How frequently in general do you use AI/ML approaches in your work or research? (n=24)

How frequently in general do you use AI/ML approaches in your work or research?	No. of respondents	% Respondents
**Not applicable**	16	40
I have used it a few times	5	12.5
I have used it many times but not very frequently	10	25
I have used it only once	2	5

Very frequently – AI/ML is the main tool I use in my work/research	7	17.5
**Total**	**40**	100

8. Please briefly describe **the purpose** of you using these AI/ML methods (e.g., the types of problems/objectives). [summary of responses with count of mentions in ()] (n=24)
    a. Data analysis: Statistical analysis, Causality between variables, interpretation of different complex, non-linear variables (5)
    b. Document/Policy analysis: Survey of literature, Autodetection of specific features (e.g. actors, targets) in law/policy documents/political statements (3)
    c. Prediction/Simulation: Modelling geographical systems to make predictions (1), physical process/extreme event simulation e.g. rainfall, wind (2), identification of key predictors (1)
    d. Impact estimation: impact modelling (monetary damages from flooding, wind) (2), health impact prediction (environmental hazards and health) (1)
    e. Policy impact analysis: Impact of drug policy scenario on resistance outcomes (1)
    f. Pattern/feature recognition and classification (2)
    g. Policy stress-testing (1)
    h. Climate resilience models (2)
    i. Groundwater process understanding (1)
    j. Disaster response: NLP on mentions of disaster topics; movement tracking, classification and segmentation for search and rescue (2)

9. *[For those who have not used AI in Q6]* Do you think the application of AI/ML methods could **be useful** to your work/study related to climate change adaptation and disaster risk management? If yes, please explain how and where it could be useful? (n=16)
    a. Yes (14)
        i. Early predictions/ warning (4)
        ii. Simpler and faster hazard modelling (1)
        iii. Satellite imagery for hazard/vulnerabilities/exposure mapping (2)
        iv. Post-disaster recovery (2)
        v. Scenario-planning and sensemaking among professionals (2)
        vi. Data-mining, digital twins (1)
        vii. Enhancing use of historical data (1)
    b. I don't know (2)

10. *[For those who have used AI in Q6]* What were the **advantages** of applying AI/ML methods in your work/research which could not be achieved otherwise? (n=24)
    a. Handling of complex/multi-variable problems and non-linear relationships that are not amenable to statistical or classical approaches (8)
    b. Handling of large quantities of data/multidimensional datasets; time-saving and efficiency from automation (5)
    c. Circumvent lack of historical data (in DRR) (1)

d. Fewer data demands (with regard to number of variables from local scale) (1)
e. Prediction of trends and future scenarios to support decision making (2)
f. Pattern recognition (1)
g. No assumptions about the used data (1)
h. Possibility to compare different approaches (1)
i. Relative ease of use of the different approaches (1)
j. Offers a different perspective (e.g. possible outcomes, influence on decisions) (1)
k. Not sure/N/A (2)

11. What, in your view, are the **challenges or limitations** that hamper the use of AI/ML methods for climate change adaptation and disaster risk management? Please elaborate where possible. (n=40)
    a. Lack of expertise/capacity/knowledge (e.g. few researchers trained in both AI/ML and DRR/environmental sciences) (8)
    b. Lack of training/historical data for AI (e.g. of extreme events with low probability); need to operate in extremes outside training data (6)
    c. Poor data availability and quality, especially socioeconomic data (e.g. health, damages) (3)
    d. Interpretability of results and models and elements (2)
    e. Difficult to extrapolate (2)
    f. Reproduces biases (2)
    g. Model definition: Difficult to define model concretely (instead using proxy variables) (1)
    h. Large amount of work needed for future projections (ABM) (1)
    i. Uncertainty of predictor variables (e.g climatic drivers) at large scales (1)
    j. Silo-ed nature of disciplines within AI (1)
    k. Demands a certain level of effort (1)
    l. Human judgement and knowledge cannot be replaced (1)
    m. Data-driven approaches should not replace statistical methods but used complementarily (1)
    n. Replication and comparison across methods (1)
    o. Moral/ethical issues (1)
    p. Maintaining impartiality and non-discrimination (1)
    q. Lack of funding (1)
    r. Lack of computational power (1)
    s. Not sure/N/A (4)

12. In your view, which of the following AI methods could support climate change adaptation and/or disaster risk management at a greater scale? (please select all that apply) (n=24)

	**No. of respondents who selected option**

1. Agent-based models and multi-agent systems	14
2. Computer Vision (e.g. image recognition)	14
3. Constraint Satisfaction and Optimisation	5
4. Data Mining	11
5. Humans and AI (e.g. Cognitive Modeling, Human AI Collaboration)	11
6. Knowledge representation and reasoning	9
7. Machine learning (e.g. Deep learning, ensemble methods)	15
8. Natural language processing	11
9. Robotics	9
10. Others (fuzzy inference, visualisation techniques)	3
11. I don't know	4

13. Please elaborate how these methods can support climate change adaptation and/or disaster risk management. [[QID13-ChoiceGroup-SelectedChoices]] (n=15)

    Technique-specific

    a. Agent-based modelling: Planning and forecasting future, Studying and prediction of actors' behavior (3), assessing policy interventions or to project how populations may adapt to new climate scenarios (1), building models that mimic reality (1), studying emergent phenomena (1)
    b. Computer vision: simulating natural processes (1), processing satellite images (1), identifying specific buildings that are vulnerable to flooding such as informal settlements (1), determining areas of risk or compare impacts before and after an event (1)
    c. Data mining: Automating tasks e.g. data collection on information about disasters (2), Combining and harmonizing existing datasets on adaptation and vulnerability (1)
    d. Humans and AI: targeted and tailored warnings before hazard events (1)
    e. Knowledge representation and reasoning: modeling of different dimensions and scales (1), prediction in complex systems (1)
    f. Machine learning: prediction of disasters, early warning (1), vulnerability analysis (1), data processing and analysis (1), Better deal with uncertainties in probabilistic forecasts (1)
    g. Natural language processing: Sentiment analysis on the acceptance of adaptation measures (1)
    h. Robotics: Future carbon sequestration (1)
    i. Fuzzy inference: offers a way to interpret changes in terms of risk
    j. Neural networks: help to model the relationship between variables for classification and prediction

    General

    a. Understanding better decision-making, behavioral processes (2)

b. simulating the diffusion processes of certain technologies (1)
c. understanding the barriers of uptake of disaster risk reduction measures (1)
d. data analysis and inference (1)
e. To gain understanding of how to implement early-warning systems/indicators or do contingency planning. (1)

14. In which of the following use-cases or application areas, based on your experience and knowledge, can AI/ML techniques be applied at a greater scale or frequency? (please select all that apply)

Option	No. of respondents who selected option	% Respondents
1. Operational disaster risk management	24	60
2. Risk finance	18	45
3. Agriculture/Food production	13	32.5
4. Transportation and critical infrastructure	18	45
5. Water governance and quality	14	35
6. Supply chain management	13	32.5
7. Climate adaptation	20	50
8. Climate vulnerability and resilience	23	57.5
9. Forest management	12	30
10. Urban heat mitigation	13	32.5
11. Adaptation behaviour and psychology	13	32.5
12. Others – technology diffusion	1	2.5
13. I don't know	5	12.5

15. Please elaborate why AI/ML techniques can be applied in the selected use-cases or application areas at a greater scale or frequency. [[QID15-ChoiceGroup-SelectedChoices]]

General Elaboration

a. They are all areas where we have a good understanding of the connection between the desired latent variable and the observable proxy
b. These fields are required to use new technology to bring sustainability
c. ML offers more tools to study the structural elements of these fields of application, for understanding their dynamics, and improve the prediction capacity.
d. items I selected are those for which the broadest coverage and time series are expected to exist.
e. Availability of data, need for preparation for uncertain and unpredictable events in the future.

f. Scenario building and understanding the consequences of systemic changes to governance structures could be useful applications in all the areas.
g. scientific understanding of these systems is very poorly understood, and in that regard, AI/ML will drive a deeper understanding of how natural (and human) systems work, which will indirectly improve overall
h. they can support ongoing processes, identify hidden complexities and help explain possible outcomes
i. There is a “Goldilocks” issue for our work where global data may not be an actionable granularity, whilst asset level engineering studies are prohibitively expensive. I would envision AI/ML to be a viable middle road to improve granularity in a cost-effective way.
j. This is a systems component that I believe AI can help us see and address. We are good at working on the parts but less sighted on the interdependencies.

Technique-Specific

k. Operational DRR: simulate complex processes, such as damage processes or evacuation; identifying damages and impacts (3), early warning systems and forecasting (2)
l. Risk finance: (Ripple) Effects in complex systems (1), support damage model applications for insurance (3)
m. Agriculture: for planning and monitoring crops (1)
n. Transport and critical infrastructure: data-driven models can be made of infrastructure black-outs in the event of climate/weather extremes (1)
o. Water governance: ML models can help assess the management of resources (2)
p. Supply chain management: Efficiency optimization (1), exploring disruption propagation in network (1), ethical/ emissions flagging (1)
q. Climate adaptation: To identify, understand and predict Adaptation Responses (2)
r. Urban heat: identifying heat islands (1), models can be built relating health outcomes to environmental variables; predictive models can be made to assess the effects of proposed urban adaptation measures (1)
s. Adaptation behavior and psychology: use of agent-based models to model human behavior (2)

Sources of data collection

The survey questions have been distributed across a variety of networks and mailing lists to ensure a broad and diverse set of respondents. These channels include:

- *SIMSOC mailing list*: A mailing list for announcements, news and discussion related to the use of computer simulation in the social sciences.
- *Risk-Kan mailing list*: Knowledge Action Network on Emergent Risks and Extreme Events
- *Network of researchers and practitioners from Zurich Flood Resilience Alliance*: https://floodresilience.net/
